# Five strategies to engage the Global South in energy transition dialogues

**DOI:** 10.1016/j.eehl.2025.100158

**Published:** 2025-05-30

**Authors:** Muhammad Salar Khan

**Affiliations:** Department of Public Policy, College of Liberal Arts, Rochester Institute of Technology, NY 14623, USA

## 1 Introduction

The escalating impacts of climate change, driven by record-breaking global temperatures and extreme weather events like floods and heatwaves, disproportionately affect the Global South. In 2024, the global average temperature reached 15.10 ​°C, 1.60 ​°C above pre-industrial levels, marking the warmest year since 1850 [1]. Despite contributing minimally to global carbon emissions [2], regions such as South Asia, Africa, and Latin America face severe climate-related disasters due to limited infrastructural resilience and adaptive capacity. Floods, in particular, have displaced millions across these regions, highlighting the urgent need for more equitable climate action.

The Paris Agreement's goal of limiting global warming to 1.5 ​°C rests on the active participation of the Global South, which represents the vast majority of the world's population. The 2024 United Nations Climate Change Conference (COP29) emphasized this urgency, setting a climate finance goal of $300 billion annually by 2035 for developing nations, though this falls short of the $1.3 trillion annually needed by 2035 for climate and nature-related goals [3]. Mainstreaming clean energy technologies into national strategies is critical for mitigating climate change, yet an equitable energy transition demands strategies tailored to the Global South's unique socio-economic contexts.

**Fig. 1:**
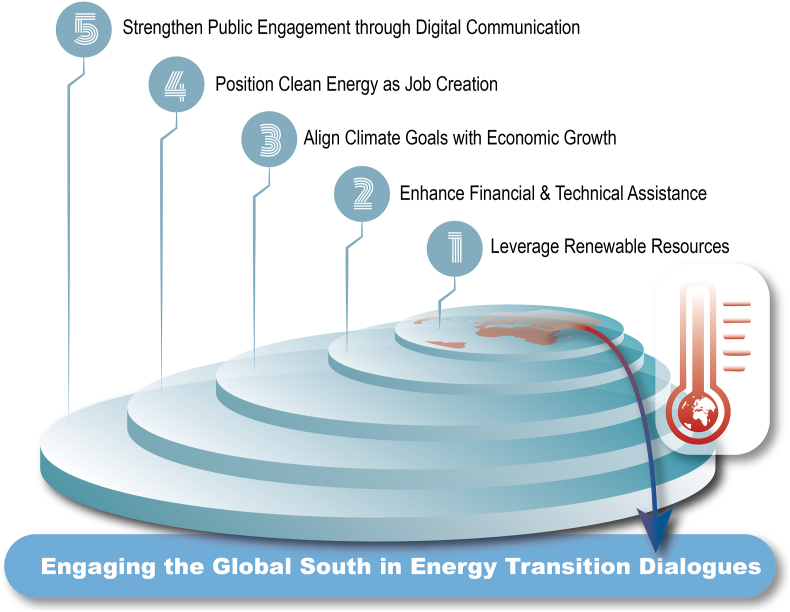
Five interlinked strategies outlined here jointly support the engagement of the Global South in energy transition dialogues. The concentric design emphasizes their shared focus on the central goal.

This policy forum addresses the question of how to effectively engage the Global South in energy transition dialogues by proposing five key strategies. It first contextualizes each strategy within existing academic and policy literature and highlights best practices from regions such as South Asia, Sub-Saharan Africa, and Latin America. It then examines policy implications, offers implementation recommendations, and identifies associated challenges. In doing so, the forum aligns these strategies with global frameworks, including the Paris Agreement and the United Nations Sustainable Development Goals (SDGs), to support inclusive and impactful climate mitigation. The strategies are listed in Fig. 1.

## 2 Strategies

The following sections outline five strategies to enhance engagement with the Global South for a smooth, inclusive energy transition.

### 2.1 Leveraging the comparative advantage of renewable resources

The Global South is richly endowed with renewable energy resources, including solar, wind, and hydropower. These regions can capitalize on this resource wealth to drive sustainable energy transitions, particularly by harnessing their comparative advantages in deploying clean technologies. Many countries in the Global South can leapfrog into newer clean energy technologies with lower risks and reduced infrastructure investments, a phenomenon often referred to as the “advantage of backwardness.” This aligns with research showing that developing countries can bypass outdated energy systems by adopting scalable renewables to meet growing energy demands sustainably [4]. Empirical evidence suggests that investments in renewable energy lead to higher emissions reductions in developing countries than in advanced economies [5].

A strategic transfer of clean technologies (such as smart grids, net metering, and decentralized solar energy solutions) can accelerate sustainable energy adoption across cities in the Global South [6]. Distributed energy resources (DERs), including mini-grids and micro-hydropower, can also expand electricity access in remote communities where centralized grids are impractical [7]. Case studies from sub-Saharan Africa and South Asia, as noted in credible reports, demonstrate that small-scale solar projects have significantly improved rural electrification, reduced fossil fuel dependence, and enhanced health outcomes [8]. For instance, Kenya's off-grid solar initiatives have electrified over 1 million rural households, while Bolivia's micro-hydropower projects have empowered remote Andean communities, both illustrating scalable models for other regions [9].

To maximize these benefits, international efforts should prioritize tailored clean technology transfers and investments in localized energy solutions. Countries such as India and Pakistan, through scalable solar projects, serve as models alongside successful micro-grid deployments in Nigeria and Kenya, offering best practices for broader adoption and replication across the Global South [10].

### 2.2 Enhancing financial and technical assistance

Literature emphasizes that effective financial and technical assistance is critical for scaling up climate action in the Global South. However, current mechanisms often fall short in addressing systemic energy challenges [11]. International organizations like the World Bank, United Nations, and regional development banks have long supported climate initiatives through financial and technical assistance, yet the growing intensity of climate disasters has raised concerns about the effectiveness of these interventions [12]. While climate finance to the Global South has increased, it remains insufficient to meet the scale of the challenge [11].

At COP29, the commitment to a $300 billion annual climate finance goal marked progress, but it still falls short of the estimated $1 trillion needed annually by 2030, and $1.3 trillion by 2035 [3]. Reports suggest that innovative financing mechanisms, such as blended finance (mixing public and private funds) and green bonds, are essential to attract private sector investment and bridge the gap [13]. In parallel, community-led initiatives and cooperative financing models can decentralize energy governance, fostering sustainability and local empowerment [14]. Notable examples include Ethiopia's community-based renewable energy cooperatives, which combine local and private funds, and Chile's green bond initiatives, which have funded urban solar projects, both offering replicable models for other regions [15].

To enhance the impact of international climate finance, efforts must focus on strengthening transparency, tailoring assistance to local contexts, and integrating climate adaptation into broader socio-economic plans [16]. Despite growing commitments, many climate strategies in the Global South remain largely theoretical, embedded in policy frameworks without tangible implementation. This gap has fueled skepticism, especially in the aftermath of ongoing climate disasters such as floods and heatwaves in Pakistan and India. In such contexts, both policymakers and local communities increasingly question the efficacy of international interventions.

Research highlights the need for collaborative frameworks that prioritize local stakeholder engagement to translate financial commitments into impactful action [14]. To rebuild trust, development partners must go beyond pledges. They must actively collaborate with local governments, climate ministries, and institutions to ensure that climate policies result in concrete, measurable outcomes on the ground.

### 2.3 Aligning climate goals with economic growth aspirations

Engaging the Global South in energy transition dialogues requires addressing the well-documented tension between climate goals and economic development priorities [17]. Many developing nations prioritize growth, infrastructure expansion, and poverty alleviation over emissions reductions. Given this reality, framing clean energy adoption through an economic lens, such as improving productivity with the same energy inputs rather than solely reducing consumption, can encourage greater buy-in from policymakers [18].

Global leadership must also acknowledge the South's legitimate developmental needs. As these countries expand infrastructure by building schools, roads, and industries, higher emissions in a business-as-usual scenario are inevitable. Studies warn that imposing external energy efficiency models without adaptation to local contexts risks resistance [19]. Climate policies should therefore be tailored to local realities and emphasize innovation and transition strategies that also drive economic growth.

One effective approach is integrating clean energy solutions into broader industrial policies. For example, Vietnam's renewable energy policies have supported its manufacturing sector, while South Africa's renewable energy program has advanced industrial development, both offering valuable lessons for other developing nations [20]. In addition, targeted subsidies and incentives for clean energy industries can encourage domestic production of solar panels, wind turbines, and battery storage solutions.

Reframing energy efficiency as “producing more outputs with the same inputs” rather than “producing more with fewer inputs”, which is more common in advanced economies, can ensure that clean energy transitions resonate more effectively within emerging economies [18]. Brazil's renewable energy sector provides a strong example of how aligning efficiency goals with industrial growth can lead to successful outcomes [21].

### 2.4 Positioning clean energy as a job creation engine

Research consistently shows that clean energy transitions can generate significant employment opportunities in the Global South, addressing key socio-economic priorities [22]. The imperative of job creation in these regions cannot be overstated. With rapidly growing populations and high unemployment rates, many countries prioritize economic expansion and labor market opportunities over climate concerns. As a result, despite their potential benefits, clean energy transitions are often sidelined in policy discussions. Global leaders can respond to this reality by framing clean energy not only as a climate solution but as a driver of employment, economic prosperity, and inclusive growth.

Emerging evidence highlights that renewable energy sectors, particularly solar and wind, create significantly more jobs per unit of energy produced than fossil fuels [23]. By positioning the clean energy transition as a tool to boost employment, attract foreign investment, and develop local skill sets, as noted in several reports [24], governments can build broader political and public support for sustainability initiatives. For instance, Brazil's wind and solar industries have created thousands of jobs across rural and semi-urban areas, while Vietnam's clean energy transition has spurred employment in its growing manufacturing sector, offering models for replication across the Global South [25].

Vocational training programs and educational initiatives can further equip local populations with the technical expertise needed for green jobs [26]. Expanding clean energy projects in rural and peri-urban areas may also alleviate urban migration pressures and promote regional economic stability, as evidenced by decentralized solar initiatives in Mali and the Philippines [27].

For developing countries, the focus should extend beyond achieving climate goals to explicitly linking clean energy policies with job creation, workforce development, and overall economic prosperity. By embedding employment-centered narratives into clean energy discussions, global leaders can ensure that sustainability efforts resonate with the immediate priorities of the Global South.

### 2.5 Strengthening public engagement through digital communication

Behavioral science highlights the value of culturally relevant and participatory communication strategies in building public engagement around climate action, particularly in the Global South [28]. Public awareness and involvement are critical for successful energy transitions, and research shows that context-sensitive messaging can significantly increase public buy-in [28].

With high smartphone penetration rates across the Global South, digital communication tools, such as interactive mobile applications, localized social media campaigns, and community-led information sessions, offer powerful platforms for fostering grassroots support for clean energy initiatives. Governments and non-profits can further use artificial intelligence and big data analytics to refine climate communication strategies, ensuring they are both targeted and effective.

Studies suggest that digital platforms providing real-time information on renewable energy incentives, weather patterns, and energy consumption trends can empower communities to make informed decisions [29]. Failing to utilize these tools can result in significant losses, as seen in the recent severe hailstorm in Islamabad, which caused millions in damages to vehicles, property, and crops due to the lack of timely public alerts. Conversely, countries like Mexico have used online platforms to support solar energy adoption, while Kenya has employed digital storytelling to build climate resilience—both demonstrating the powerful potential of digital engagement in diverse contexts [30,31].

To amplify these benefits, the global community must invest in the necessary infrastructure, training, and communication technologies that equip the Global South with scalable and accessible climate awareness tools supported by proven digital engagement strategies.

## 3 Policy implications

The global fight against climate change requires a unified, inclusive approach that places the Global South, home to over 80% of the world's population, at the center of energy transition dialogues. Recent extreme weather events, such as devastating floods in Nepal and Brazil and unprecedented heatwaves across Asia, highlight the disproportionate burden faced by these regions despite their minimal contribution to global carbon emissions. By 2050, an estimated 143 million people in the Global South may be displaced due to climate change [32], further intensifying migration pressures and negatively impacting both physical and mental health [33]. Achieving the Paris Agreement's target of limiting global warming to 1.5 ​°C and cutting emissions in half by 2030 depends on actively engaging these regions through strategies tailored to their specific socio-economic and infrastructural realities.

To support an equitable energy transition, this policy forum proposes five strategies that also align with the United Nations SDGs, particularly SDG 7 (Affordable and Clean Energy), SDG 8 (Decent Work and Economic Growth), and SDG 13 (Climate Action).

1) The Global South's rich renewable energy potential, such as solar and wind, can be harnessed through public-private partnerships and tax incentives for clean technology transfers. Kenya's off-grid solar projects, which have brought electricity to millions, illustrate this approach.
2) Enhancing financial and technical assistance requires scaling up innovative instruments like green bonds and community-led models. Ethiopia's energy cooperatives and Chile's urban solar initiatives, supported by transparent funding mechanisms, offer replicable examples.
3) Aligning climate goals with economic development involves integrating clean energy into national industrial strategies. Countries like Vietnam and South Africa have used subsidies to promote renewable energy manufacturing while framing energy efficiency as a path to productivity.
4) Positioning clean energy as a job creation engine means investing in vocational training and incentivizing rural projects. Brazil's solar sector and initiatives in Mali and the Philippines demonstrate how renewable energy can generate employment and ease urban migration.
5) Strengthening public engagement through digital communication tools like interactive platforms and localized social media campaigns can help build grassroots support. Mexico's use of digital tools for solar adoption and Kenya's digital climate storytelling show how strategic communication fosters public participation.

These strategies also directly contribute to global frameworks. As examples, Bolivia's micro-hydropower systems expand rural energy access (SDG 7), Brazil's solar industry creates green jobs (SDG 8), and transparent climate finance (reinforced at COP29) supports climate adaptation in many vulnerable regions (SDG 13). Nonetheless, major implementation challenges remain. Bureaucratic inefficiencies, limited technical expertise, and competing priorities such as poverty reduction often stall progress. For instance, while India's solar program has achieved notable success, replicating such efforts in Sub-Saharan Africa will require targeted infrastructure investment, regulatory reform, and international collaboration.

With most people at high risk of flooding living in Sub-Saharan Africa, and with growing threats from hurricanes and droughts, urgent, scalable action is critical. Policymakers must prioritize regional renewable energy hubs, cross-border technology collaboration, innovative financing mechanisms such as green bonds, and the use of artificial intelligence to enhance climate communication, including targeted digital campaigns. Embedding these efforts within the frameworks of the Paris Agreement and the Sustainable Development Goals provides a pathway to lower emissions, build greater climate resilience, stimulate job creation, and elevate the Global South's priorities in energy transition dialogues. Only through inclusive and locally informed strategies can the world achieve a just and sustainable energy future.

## CRediT authorship contribution statement

**Muhammad Salar Khan:** Writing – review & editing, Writing – original draft, Supervision, Investigation, Formal analysis, Conceptualization.

## Declaration of competing interests

The author has nothing to declare.
